# Aging and Sex Differences in Brain Volume and Cerebral Blood Flow

**DOI:** 10.14336/AD.2023.1122

**Published:** 2024-10-01

**Authors:** Hiroto Kawano, Shigeki Yamada, Yoshiyuki Watanabe, Satoshi Ii, Tomohiro Otani, Hirotaka Ito, Ko Okada, Chifumi Iseki, Motoki Tanikawa, Shigeo Wada, Marie Oshima, Mitsuhito Mase, Kazumichi Yoshida

**Affiliations:** ^1^Department of Neurosurgery, Shiga University of Medical Science, Shiga, Japan.; ^2^Department of Neurosurgery, Nagoya City University Graduate School of Medical Science, Aichi, Japan.; ^3^Interfaculty Initiative in Information Studies / Institute of Industrial Science, The University of Tokyo, Tokyo, Japan.; ^4^Department of Radiology, Shiga University of Medical Science, Shiga, Japan.; ^5^Faculty of System Design, Tokyo Metropolitan University, Tokyo, Japan.; ^6^Department of Mechanical Science and Bioengineering, Graduate School of Engineering Science, Osaka University, Osaka, Japan.; ^7^Medical System Research & Development Center, FUJIFILM Corporation, Tokyo, Japan.; ^8^Department of Behavioral Neurology and Cognitive Neuroscience, Tohoku University Graduate School of Medicine, Sendai, Miyagi, Japan.; ^9^Division of Neurology and Clinical Neuroscience, Department of Internal Medicine III, Yamagata University School of Medicine, Yamagata, Japan.

**Keywords:** aging, sex difference, regional brain volume, cerebral blood flow, circle of Willis

## Abstract

How do regional brain volume ratios and cerebral blood flow (CBF, mL/min) change with aging, and are there sex differences? This study aimed to comprehensively evaluate the relationships between regional brain volume ratios and CBF in healthy brains. The study participants were healthy volunteers who underwent three-dimensional T1-weighted MRI, time-of-flight MR angiography, and four-dimensional (4D) flow MRI between 2020 and 2022. The brain was automatically segmented into 21 brain subregions from 3D T1-weighted MRI, and CBF in 16 major intracranial arteries were measured by 4D flow MRI. The relationships between segmented brain volume ratios and CBFs around the circle of Willis were comprehensively investigated in each decade and sex. This study included 129 healthy volunteers (mean age ± SD, 48.2 ± 16.8; range, 22-92 years; 43 males and 86 females). The association was strongest between the cortical gray matter volume ratio and total outflow of the intracranial major arteries distal to the circle of Willis (Pearson’s correlation coefficient, *r*: 0.425). In addition, the mean flow of the total inflow and outflow around the circle of Willis were significantly greater in women than men, and significant left-right differences were observed in CBFs even on the peripheral side of the circle of Willis. Moreover, the correlation was strongest between the left cortical gray matter volume ratio and the combined flows of the left anterior and posterior cerebral arteries distal to the circle of Willis (*r*: 0.486). There was a trend toward greater total intracranial CBF, especially among women in their 40s and younger, who had a larger cortical gray matter volume. This finding may be one of the reasons for the approximately twofold higher incidence of cerebral aneurysms and subarachnoid hemorrhage, and a threefold higher incidence of migraine headaches.

## INTRODUCTION

The brain volume [1-4], and its association with the cerebral blood flow (CBF) [5-14], are gradually decreased with normal aging. Furthermore, patients with dementia have not only decreased pathological regional brain volume [15-17], but also abnormally decreased regional CBF [18-20]. In addition, quantitative assessment of CBF has been highlighted as the most important predictor of clinical outcome in patients with acute cerebral infarction due to the main cerebral artery occlusion [21, 22]. Nuclear medicine examinations, such as ^15^O-labeled water or gas-inhalation positron emission tomography [10, 23], and ^123^I-iodoamphetamine single-photon emission computed tomography [24, 25], were thought to be gold-standard imaging modalities used to estimate CBFs and collateral circulations; however, their resolution is lower than that of MRI sequences. Recently, the phase-contrast (PC) MRI and arterial spin labeling (ASL) MRI sequences have become widely used because it is a minimally invasive and simple method that quantifies CBF [5-9, 12-14]. Moreover, 3D PC MRI, namely 4D flow MRI [11, 19], has made it possible to measure 3D flow velocities of intracranial cerebral arteries with complex bends, twists, and branches. Although the relationship between the total brain volume and total CBFs and aging in healthy participants has been reported [6, 14, 26], no study has evaluated the relationship between regional brain volume and CBF. We recently reported on the effects of aging and sex differences in volume ratios of the brain region and cerebrospinal fluid (CSF) spaces in healthy volunteers using the deep learning model [27]. In addition, the CBFs in intracranial arteries around the circle of Willis of the same healthy participants were measured on 4D flow MRI. We hypothesized that cortical gray matter, which linearly declines with normal aging, may have a stronger correlation with CBF, compared with subcortical gray and white matter. Therefore, this study aimed to identify the location and left-right differences in the brain subregion that were mostly associated with age-related decreases in CBFs of intracranial arteries around the circle of Willis in healthy volunteers. In addition, we also aimed to investigate the sex differences in regional brain volume ratios and CBFs in normal aging.

## MATERIALS AND METHODS

### Study population

Details of data collection, anonymization, image acquisition, and data processing methods of automatic brain segmentation and 4D flow MRI were described in our previous study [27, 28]. In brief, healthy medical staff volunteers and their family members aged ≥20 years were recruited by open recruitment from November 2020 to April 2022. The study design and protocol of this prospective and observational study were approved by the ethics committees for human research at our institutes (IRB Number: 60-22-0083, R2019-227). All participants underwent MRI examinations for research purposes after obtaining written consent. MRI data were extracted within the hospital where the imaging took place, ensuring the anonymization of personal information in a linkable manner. Researchers utilized the MRI data with only age and sex information, preventing the identification of individuals. The inclusion criteria for this study were as follows: participants must be 20 years of age or older, have no history of brain injury, brain tumor, or cerebrovascular disease on the previous brain MRI or those who had never undergone brain CT or MRI and had no neurological symptoms. The exclusion criteria were as follows: participants with large artifacts in the head, particularly dentures, which affected the MRI results. None had asymptomatic cerebral infarction or stenosis/occlusion of the main intracranial arteries on MRI. From the 133 healthy volunteers (mean age, 43.9 ± 14.7; range, 21-92 years; 46 males and 87 females) in our previous study [27], four were excluded because 4D flow MRI for the intracranial arteries including the circle of Willis was not performed.

### Image acquisitions

All MRI examinations were performed using a 3-tesla MRI machine (Signa Architect 3.0T or Discovery MR 750W, GE Healthcare, Milwaukee, WI, USA). The sequence parameters of 3D T1-weighted magnetization prepared rapid gradient echo (MPRAGE) were as follows: repetition time (TR), 2471 ms; echo time (TE), 3.13 ms; inversion time, 1000 ms; flip angle (FA), 8°; matrix 256 × 256; voxel size, 0.9 × 0.9 × 0.9 mm; and acquisition time, approximately 4 min; those of time-of-flight MR angiography were as follows: TR,254 ms; TE, 3.3 ms; flip angle, 15°; matrix, 512 × 512; voxel size, 0.391 × 0.391 × 0.98 mm; and acquisition time, approximately 4 min.

The time-resolved 3D velocity encoding data obtained from the 4D flow MRI sequence with 120 cm/s of velocity encoding and synchronizing the peripheral pulse rate were measured from the finger pulse oximeter (TR, 6.24 msec; TE, 3.02 msec; FA, 8°; field of view, 200 mm; 256 × 256; voxel size, 0.703 × 0.703 × 0.972 mm; number of cardiac phases, 12; and acquisition time, approximately 10 min). The image range of 4D flow MRI was obtained in the oblique plane with a width of 35 mm (36 slices), including bilateral internal carotid arteries (ICAs), basilar artery (BA), A1 and A2 portions of bilateral anterior cerebral arteries (ACAs), M1 portion of bilateral middle cerebral arteries (MCAs), P1 and P2 portions of bilateral posterior cerebral arteries (PCAs), anterior communicating artery (ACoA), and bilateral posterior communicating arteries (PCoAs).

### Brain segmentation process

From the 3D T1-weighted MPRAGE sequence, the brain was automatically segmented into the following 21 brain subregions within 1 min: the frontal cortex; parietal cortex; temporal cortex; occipital cortex; insular cortex; cerebral white matter; hippocampus, including the parahippocampal gyrus (entorhinal cortex); basal ganglia, including the caudate nucleus; putamen; globus pallidus; the limbic system, including the cingulate gyrus and amygdala; brainstem, including the thalamus, hypothalamus, midbrain, pons, and medulla oblongata; and cerebellum using the Brain Subregion Analysis application on an independent 3D volume analyzer workstation (SYNAPSE 3D; FUJIFILM Corporation, Tokyo, Japan) by referring to our previous study for the details of the method and reliability (Video 1) [27]. In this study, the cortical gray matter was defined as the combined region of the frontal, temporal, parietal, and occipital cortices, and subcortical gray matter was defined as the combined region of the hippocampus, basal ganglia, and brainstem. Volume ratios of the segmented regions divided by their intracranial volume.

### Four-dimensional flow analysis process

**Figure 1.**
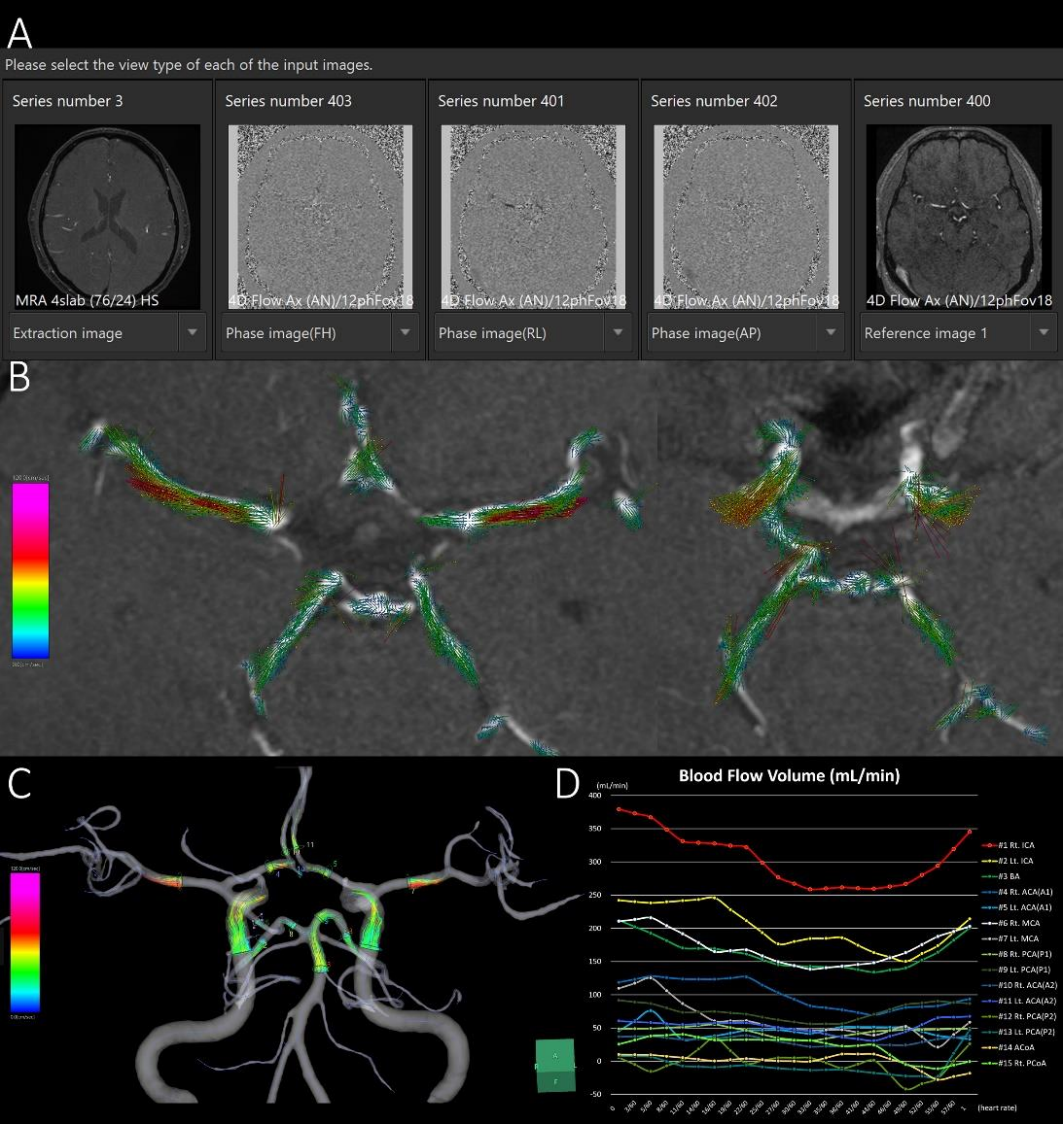
Schematic diagram of 4D flow MRI analysis in representative healthy middle-aged participants. The phase-contrast (PC) images on the tri-axial direction and magnitude images of 4D flow MRI are integrated with high-resolution time-of-flight MR angiography (A). The PC images on each tri-axial direction provide the flow velocity distribution of the direction of the magnetic field gradient. The 3D flow vector and its motion in one cardiac cycle are visualized on the 4D flow application. The flow velocity vectors on the 2D display (B) and streamlines on the 3D display (C) at 15 ROIs are shown. (D) the x-axis is the division time during one cardiac cycle, and the y-axis is the blood flow volume (mL/min).

For 4D flow analysis, 3D velocity encoding data obtained from tri-axial PC images of 4D flow MRI and morphological data of the major cerebral arteries including the circle of Willis obtained from the time-of-flight MR angiography were combined on the 4D flow application in the SYNAPSE 3D workstation (Fig. 1 and Video 2).

**Figure 2.**
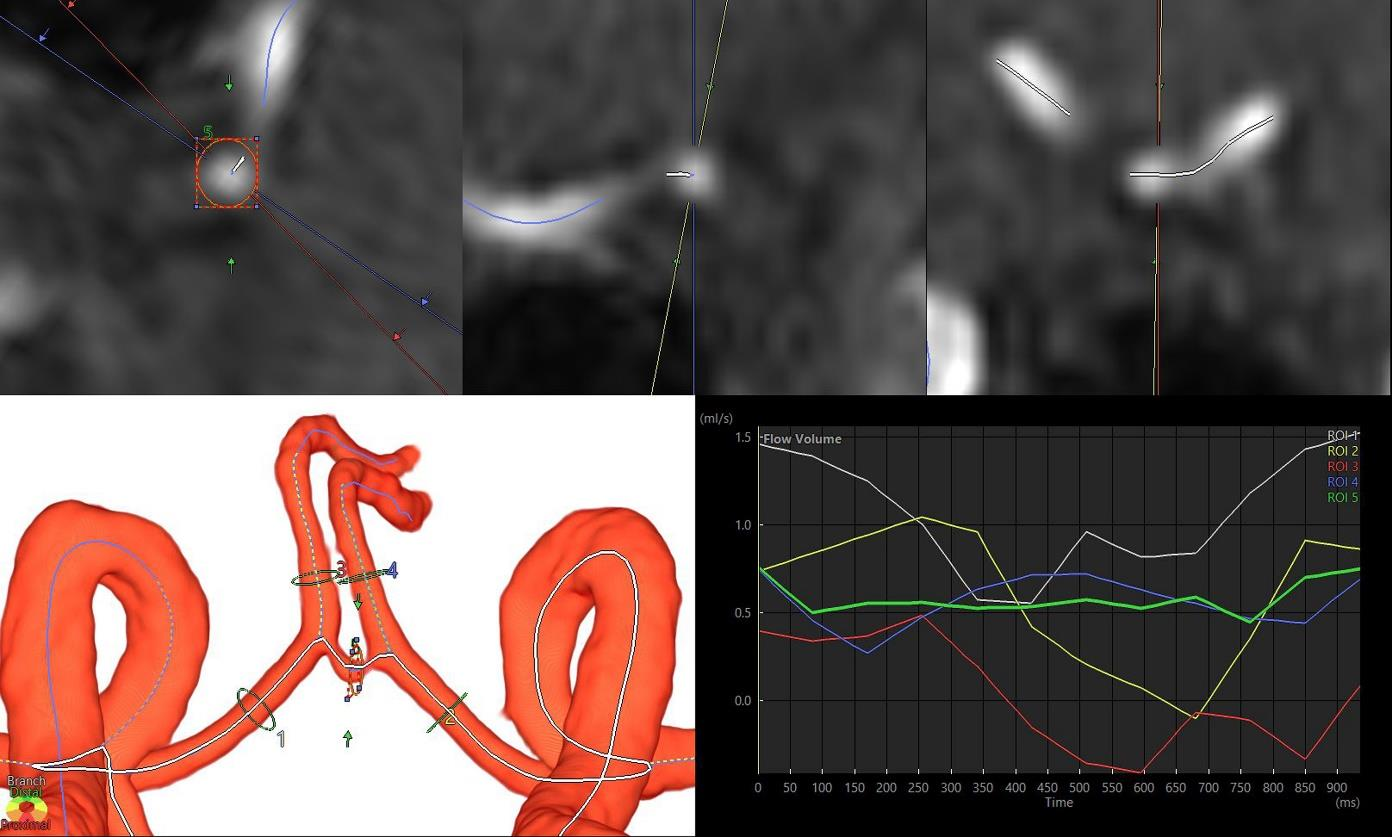
Cross-sectional region of interest (ROI) for flow velocity measurement in bilateral anterior cerebral arteries (ACAs) and anterior communicating artery (ACoA). The upper three figures are 2D oblique views of ACoA, with a cross section orthogonal to the centerline automatically generated on the left. The 3D volume-rendering image in the lower left depicts the automatically generated centerline after extraction of the circle of Willis. The line graph in the lower right shows the change in flow volume in the five ROIs during one cardiac cycle.

After the segmentation of major cerebral arteries, centerlines of the segmented cerebral arteries were automatically calculated using thinning and nonuniform rational B-splines curves. On the automatically generated perpendicular cross-section to the centerline of the target artery, a circle of the region of interest (ROI) was manually drawn around the vessel lumen boundary (Fig. 2).

The CBF of each artery (mL/min) was obtained by multiplying the mean flow velocity in the ROI by the area of the ROI. CBFs were measured at 16 ROIs: BA, ACoA, and right and left ICAs, ACAs (A1), ACAs (A2), MCAs, PCAs (P1), PCAs (P2), and PCoA. An invisible ACoA or PCoA indicates no flow velocity data (missing values). In arteries with slow flow, such as ACoA in Figure 2, three ROIs were taken in the same segment to confirm that the flow was stable, and the ROI with the flow velocity near the median was selected from the three ROIs. The total inflow (mL/min) was defined as combined CBFs of the bilateral ICAs and BA; the total outflow #1 was defined as the combined CBFs of the A1 portion of bilateral ACAs, bilateral MCAs, and P1 portions of bilateral PCAs; and the total outflow #2 was defined as combined CBFs of the A2 portion of bilateral ACAs, bilateral MCAs, and P2 portions of bilateral PCAs.

### Statistical analysis

The volunteers were divided into the following six subgroups based on their ages at the time of MRI examination: 20s, 30s, 40s, 50s, 60s, and ≥70 years. The Kruskal-Wallis rank sum test was used to compare the mean ± standard deviation (SD) volume ratios of the segmented brain and CBF of the arterial ROI in six age subgroups. The Mann-Whitney-Wilcoxon test was performed to compare sex differences and right-left differences in the segmented volume ratios and CBFs of the intracranial major arteries. The chi-square test was used to compare the proportions of the groups. The relationships between segmented volume ratios and CBFs were comprehensively investigated using Pearson’s correlation coefficient (*r*) and 95% confidence intervals (CIs). Statistical significance was assumed at a probability (*P*) value of <0.05. All missing data points were treated as deficit data that did not affect other variables. Statistical analyses were performed using R (version 4.2.3; The R Foundation for Statistical Computing; http://www.R-project.org).

**Table 1:** Mean ± SD blood flow volume of intracranial artery by decade.

	20s	30s	40s	50s	60s	70<	*P*-value
Total number (Female:Male)	21 (15:6)	22 (15:7)	24 (14:10)	25 (20:5)	22 (15:7)	15 (7:8)	
Left ICA	370 ± 91	356 ± 109	347 ± 113	384 ± 107	276 ± 113	310 ± 105	0.018
Right ICA	351 ± 100	348 ± 132	334 ± 78	340 ± 111	292 ± 154	312 ± 107	0.376
BA	205 ± 58	171 ± 68	196 ± 61	157 ± 63	128 ± 74	129 ± 45	<0.001
Left ACA (A1)	129 ± 42	133 ± 46	112 ± 56	139 ± 63	91 ± 44	96 ± 37	0.010
Right ACA (A1)	121 ± 60	108 ± 45	97 ± 38	95 ± 37	86 ± 53	95 ± 43	0.201
Left MCA	187 ± 41	164 ± 53	171 ± 47	174 ± 52	140 ± 65	165 ± 66	0.092
Right MCA	218 ± 59	177 ± 48	203 ± 68	184 ± 49	164 ± 77	162 ± 54	0.093
Left PCA (P1)	109 ± 30	75 ± 32	90 ± 33	86 ± 33	70 ± 39	72 ± 32	0.008
Right PCA (P1)	105 ± 39	101 ± 40	104 ± 48	86 ± 51	73 ± 38	60 ± 29	0.025
Left ACA (A2)	130 ± 56	106 ± 33	92 ± 28	102 ± 34	68 ± 30	73 ± 32	<0.001
Right ACA (A2)	104 ± 33	93 ± 38	95 ± 34	82 ± 31	67 ± 34	73 ± 36	0.003
Left PCA (P2)	121 ± 41	88 ± 37	101 ± 41	98 ± 34	66 ± 35	66 ± 27	<0.001
Right PCA (P2)	127 ± 36	101 ± 40	102 ± 27	108 ± 33	65 ± 37	76 ± 27	<0.001
ACoA	32 ± 36	26 ± 33	36 ± 36	41 ± 27	37 ± 35	10 ± 6	0.087
Left PCoA	17 ± 9	66 ± 69	48 ± 31	68 ± 28	47 ± 34	31 ± 35	0.011
Right PCoA	41 ± 40	69 ± 64	51 ± 30	85 ± 29	69 ± 38	72 ± 41	0.099
Total inflow volume	925 ± 201	875 ± 243	878 ± 190	881 ± 206	696 ± 276	751 ± 206	0.045
Total outflow volume #1	860 ± 189	746 ± 201	788 ± 213	761 ± 194	595 ± 231	581 ± 213	0.018
Total outflow volume #2	896 ± 178	748 ± 209	757 ± 187	742 ± 175	568 ± 244	614 ± 193	0.001
Left total outflow volume #1	426 ± 79	369 ± 94	374 ± 105	392 ± 118	296 ± 127	332 ± 114	0.022
Right total outflow volume #1	439 ± 140	389 ± 119	409 ± 127	363 ± 106	332 ± 157	303 ± 110	0.110
Left total outflow volume #2	454 ± 110	370 ± 102	362 ± 97	373 ± 93	273 ± 119	304 ± 109	<0.001
Right total outflow volume #2	454 ± 98	372 ± 116	396 ± 101	373 ± 90	295 ± 130	311 ± 97	0.001

*P*-value, probability value for the Kruskal-Wallis test among the three age groups Total inflow, combined CBFs of bilateral ICAs and BA Total outflow #1, combined CBFs of bilateral ACAs (A1), MCAs, and PCAs (P1) Total outflow #2, combined CBFs of bilateral ACAs (A2), MCAs, and PCAs (P2)

## RESULTS

### Clinical characteristics

**Figure 3.**
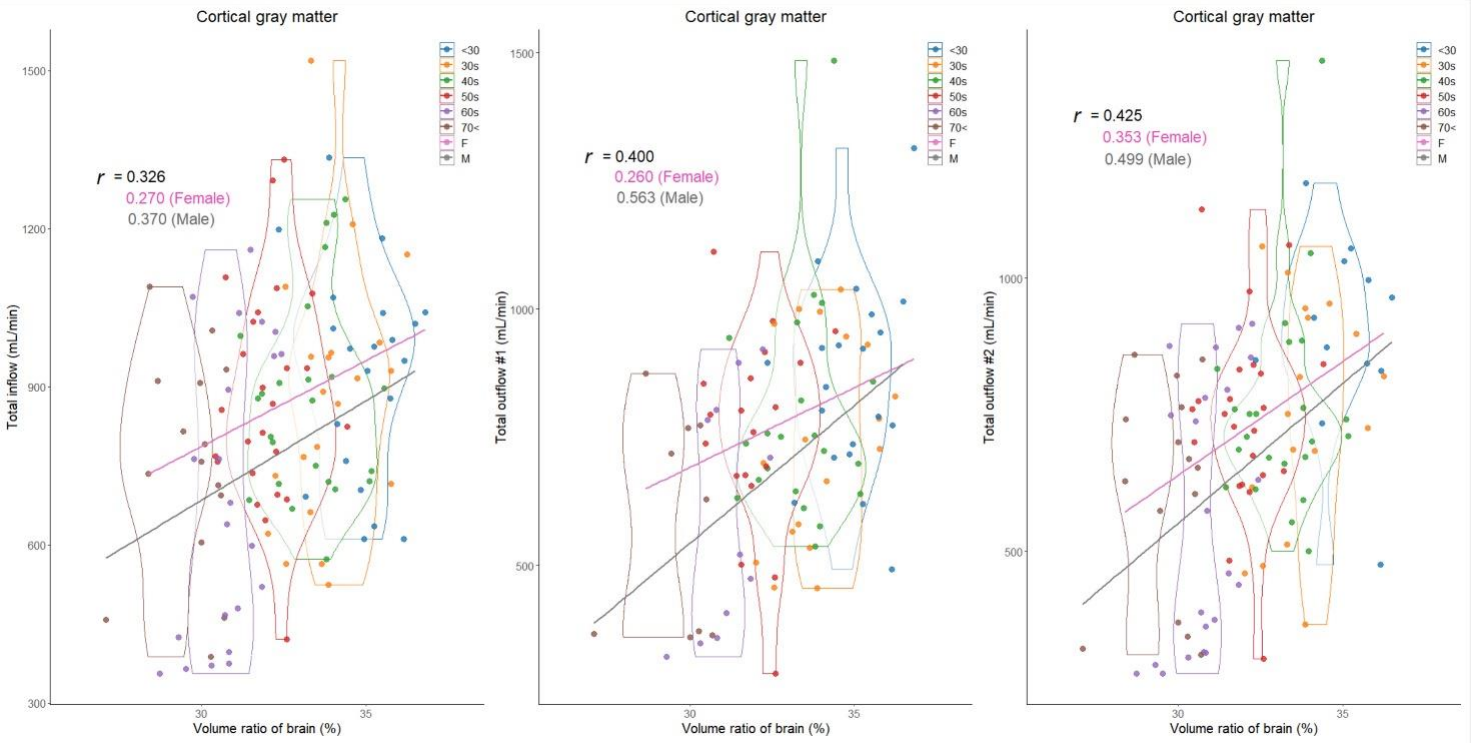
Distribution of cortical gray matter volume ratios (%) and intracranial blood flow (mL/min) classified by age and sex. Violin plots show the distribution in each age group and straight lines indicate linear regression equations by sex. The y-axis of the left scatter plot shows the total inflow, which was defined as combined blood flows of bilateral ICAs and BA; that of the middle shows the total outflow #1, which was defined as combined blood flows of bilateral ACAs (A1), MCAs, and PCAs (P1); and that of the right shows the total outflow #2, which was defined as combined blood flows of bilateral ACAs (A2), MCAs, and PCAs (P2). Color coding indicates age and sex classifications as shown in the legend.

**Figure 4.**
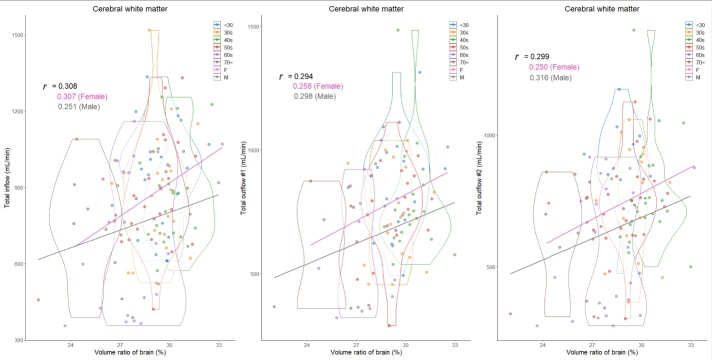
Distribution of cerebral white matter volume ratios (%) and intracranial blood flow (mL/min) classified by age and sex. Violin plots show the distribution in each age group and straight lines indicate linear regression equations by sex. The y-axis of the left scatter plot shows the total inflow, which was defined as combined blood flows of bilateral ICAs and BA; that of the middle shows the total outflow #1, which was defined as combined blood flows of bilateral ACAs (A1), MCAs, and PCAs (P1); and that of the right shows the total outflow #2, which was defined as combined blood flows of bilateral ACAs (A2), MCAs, and PCAs (P2). Color coding indicates age and sex classifications as shown in the legend.

**Figure 5.**
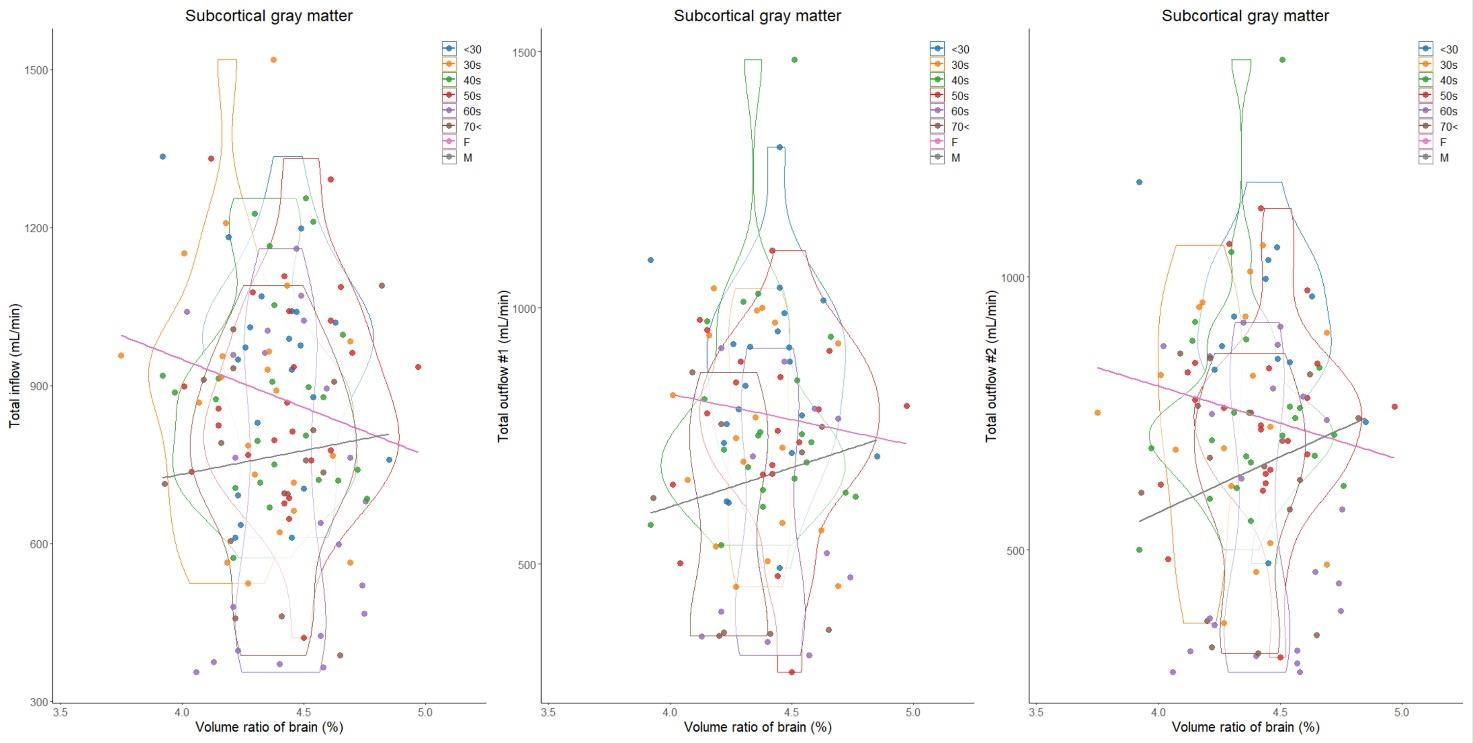
Distribution of subcortical gray matter volume ratios (%) and intracranial blood flow (mL/min) classified by age and sex. Violin plots show the distribution in each age group and straight lines indicate linear regression equations by sex. The y-axis of the left scatter plot shows the total inflow, which was defined as combined blood flows of bilateral ICAs and BA; that of the middle shows the total outflow #1, which was defined as combined blood flows of bilateral ACAs (A1), MCAs, and PCAs (P1); and that of the right shows the total outflow #2, which was defined as combined blood flows of bilateral ACAs (A2), MCAs, and PCAs (P2). Color coding indicates age and sex classifications as shown in the legend.

**Figure 6.**
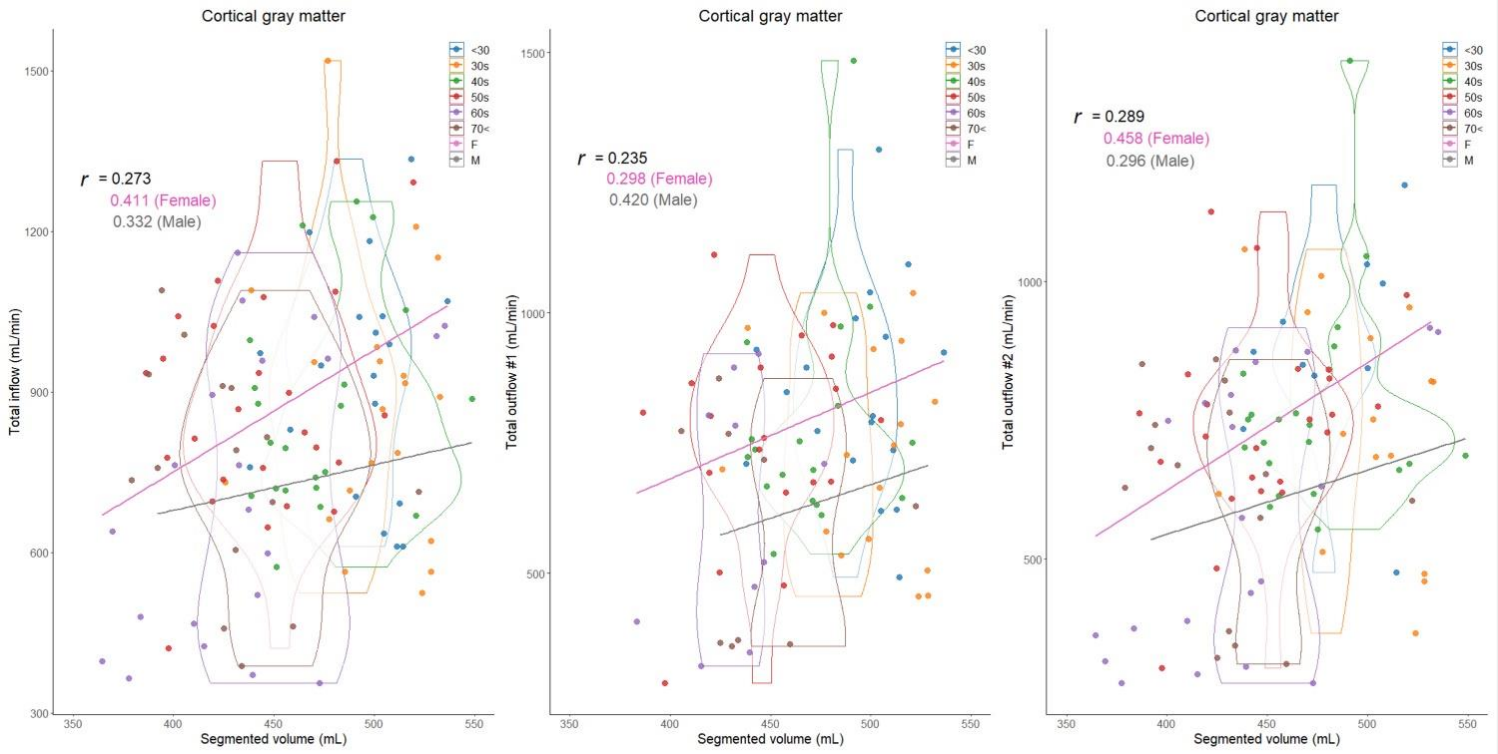
Distribution of cortical gray matter volume (mL) and intracranial blood flow (mL/min) classified by age and sex. Violin plots show the distribution in each age group and straight lines indicate linear regression equations by sex. The y-axis of the left scatter plot shows the total inflow, which was defined as combined blood flows of bilateral ICAs and BA; that of the middle shows the total outflow #1, which was defined as combined blood flows of bilateral ACAs (A1), MCAs, and PCAs (P1); and that of the right shows the total outflow #2, which was defined as combined blood flows of bilateral ACAs (A2), MCAs, and PCAs (P2). Color coding indicates age and sex classifications as shown in the legend.

In total, 129 healthy volunteers (mean age ± SD, 48.2 ± 16.8; range, 22-92 years; 43 males and 86 females) were included. Because the intracranial volume was significantly different between males (mean volume ± SD, 1531.2 ± 137.3 mL) and females (1393.3 ± 91.7 mL), volume ratios of the segmented regions were used in this study. The mean CBFs of bilateral ICAs demonstrated a less pronounced decrease with aging, whereas that of BA exhibited a significant decrease (Table 1). The CBF of the bilateral MCAs (M1) was the least affected by aging, with only about a 20% decrease in CBF, and CBF was maintained even at age 70 or older. The ACA (A1) and PCA (P1) showed about a 30% reduction in CBF, the ACA (A2) exhibited approximately a 40% reduction in CBF, and the PCA (P2) was the most susceptible to aging, with about a 50% reduction in CBF (Table 1). Therefore, the mean CBF of total outflow #2 exhibited the most significant decreasing trend with aging, followed by that of total outflow #1. As shown in Figure 3, the mean volume ratio of the cortical gray matter linearly decreases with aging, whereas that of the cerebral white matter was the most increased in those in their 40s (Figure 4). Moreover, the association was strongest between the cortical gray matter volume ratio and total outflow #2 (*r*: 0.425; 95% CI: 0.260-0.566; *P*, <0.001) (Fig. 3), whereas the cerebral white matter was moderately associated with any total CBFs (*r*: <0.308) (Fig. 4). In addition, the volume ratio of the subcortical gray matter was less affected by aging and not associated with any CBFs (*r* <0.04) (Fig. 5). The relationships between the distribution of cortical gray matter volume (mL) and the total inflow and outflow of CBF (mL/min) were also examined, considering the influence of the intracranial volume of the participants. As shown in Figure 6, the participants were categorized into two groups: those in their 40s and younger and those in their 50s and older. The younger group (40s and younger) exhibited a greater cortical gray matter volume than the older group. However, there was more variability in the total outflow #1 and #2 than in cortical gray matter volume.

Mean outflow #1 and #2 in the younger group were 800 ± 203 and 786 ± 198 mL/min, whereas they were 672 ± 222 and 647 ± 218 mL/min in the older group, respectively, which were significantly lower than those in the younger group.

### Sex difference in the segmented volume ratio and cerebral blood flow

Overall, the total CBFs in proportion to the segmented brain volume ratios were higher in women than in men. As shown in Figures 3 through 6 and Table 2, besides the effects of aging, women have significantly higher total inflow and total outflow #1 and #2 than men. On the basis of cortical gray matter volume without accounting for the influence of intracranial volume, sex differences were more pronounced, especially in the younger group (Figure 6). Healthy women had significantly higher mean CBFs of bilateral ICAs, right ACA (A2), and bilateral PCA (P2) than healthy men (Table 2). Because both segmented brain volume ratios and CBFs of intracranial arteries were strongly affected by aging, sex differences in these relationships with aging were further investigated. In the younger group (40s and younger), women had significantly higher total inflow (934 ± 219 mL vs. 813 ± 168 mL, *P* = 0.014) and total outflow #2 (831 ± 204 mL vs. 695 ± 154 mL, *P* = 0.011) than men. In the older group (50s and older), women exhibited a significantly higher total outflow #1 than men (673 ± 213 mL vs. 594 ± 224 mL, *P* = 0.020).

**Table 2:** Sex differences in the mean blood flows of intracranial arteries.

	Total	Female	Male	*P* value
Total number (Female : Male)	129	86	43	
Mean age ± SD	48.2 ± 16.8	47.0 ± 16.0	50.8 ± 18.1	0.274
Left ICA	342.8 ± 111.1	360.3 ± 109.7	308.3 ± 107.0	0.012
Right ICA	330.7 ± 115.7	348.0 ± 116.0	296.5 ± 108.5	0.013
BA	166.0 ± 68.3	169.2 ± 73.8	159.6 ± 56.2	0.387
Left ACA (A1)	118.3 ± 52.5	123.9 ± 50.6	107.2 ± 55.1	0.073
Right ACA (A1)	100.6 ± 46.7	106.8 ± 45.9	88.6 ± 46.3	0.026
Left MCA	167.1 ± 54.8	172.8 ± 54.2	155.8 ± 54.8	0.153
Right MCA	185.8 ± 62.3	190.9 ± 62.9	175.9 ± 60.6	0.119
Left PCA (P1)	85.0 ± 35.3	88.0 ± 36.9	79.3 ± 31.6	0.167
Right PCA (P1)	91.5 ± 44.4	93.2 ± 47.7	88.2 ± 37.8	0.556
Left ACA (A2)	96.0 ± 41.2	101.0 ± 43.0	86.1 ± 35.9	0.067
Right ACA (A2)	86.0 ± 35.8	91.5 ± 37.5	75.2 ± 29.8	0.017
Left PCA (P2)	88.9 ± 39.7	97.8 ± 42.0	70.7 ± 26.9	<0.001
Right PCA (P2)	96.1 ± 38.5	102.3 ± 38.9	83.0 ± 34.5	0.014
ACoA	32.1 ± 32.4	29.9 ± 30.8	36.4 ± 35.6	0.478
Left PCoA	48.8 ± 42.1	51.8 ± 45.3	37.9 ± 26.9	0.558
Right PCoA	62.8 ± 41.6	59.7 ± 37.9	73.6 ± 52.8	0.318
Total inflow	839.5 ± 232.5	877.5 ± 235.3	764.4 ± 209.8	0.008
Total outflow #1	749.0 ± 218.7	791.7 ± 219.2	668.4 ± 196.5	0.006
Total outflow #2	711.4 ± 219.7	746.4 ± 222.2	640.5 ± 199.2	0.015
Left total outflow #1	369.5 ± 111.7	383.0 ± 110.4	343.6 ± 110.9	0.075
Right total outflow #1	382.0 ± 131.4	401.4 ± 134.5	346.5 ± 119.3	0.034
Left total outflow #2	350.0 ± 115.5	369.7 ± 116.9	309.9 ± 102.6	0.015
Right total outflow #2	365.7 ± 116.0	382.7 ± 118.9	329.9 ± 102.4	0.012

*P*-value, prevalence ratio for the Wilcoxon rank sum test.

### Laterality of age-related changes in the segmented volume ratio and cerebral blood flow

Segmented volume ratios in the cortical and subcortical gray matter, frontal, occipital, and insula cortices, limbic system, hippocampus, and basal ganglia had statistically significant left-right differences (Table 3). No significant difference was observed between the CBFs of the left and right ICAs, whereas the right MCA (M1) and left ACA (A1) had significantly higher CBFs, compared to the opposite side.

**Figure 7.**
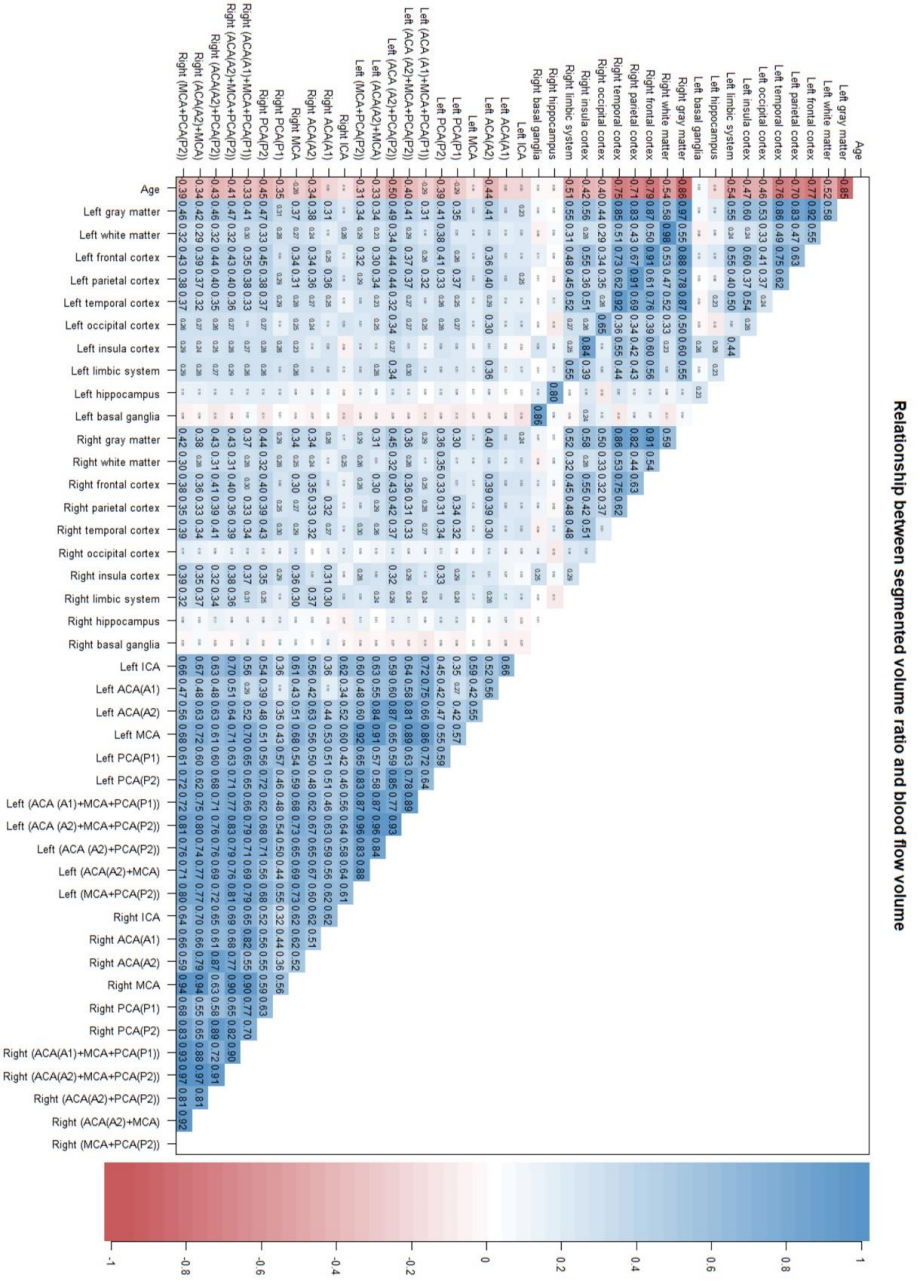
Comprehensive correlation analysis between left and right segmented volume ratios and intracranial blood flows. A comprehensive correlation analysis of left and right segmented volume ratios and intracranial blood flow volumes measured by 4D flow MRI with age is presented. As shown in the color bar on the right, the blue gradient indicates the strength of the positive correlation, whereas the red gradient indicates the strength of the negative correlation. In addition, the numbers written inside were Pearson’s correlation coefficients.

Figure 7 shows the comprehensive investigation of the relationship between segmented volume ratios and CBFs for the left and right sides. The volume ratios in the same segmented regions were strongly correlated on the left and right sides. In addition, the CBF of each artery and their combinations were also strongly correlated on the left and right sides. However, compared to the right side, the correlations of the left cortical gray matter volume ratio with the CBFs in the left cerebral arteries were stronger. The correlation was strongest between the left cortical gray matter volume ratio and combined CBFs of the left ACA (A2) and left PCA (P2) (*r*: 0.486; 95% CI: 0.331-0.616; *P* < 0.001), whereas the CBF in left MCA was weakly correlated with the left cortical gray matter volume ratio (*r*: 0.217; 95% CI: 0.045-0.376; *P*, 0.014).

**Figure 8.**
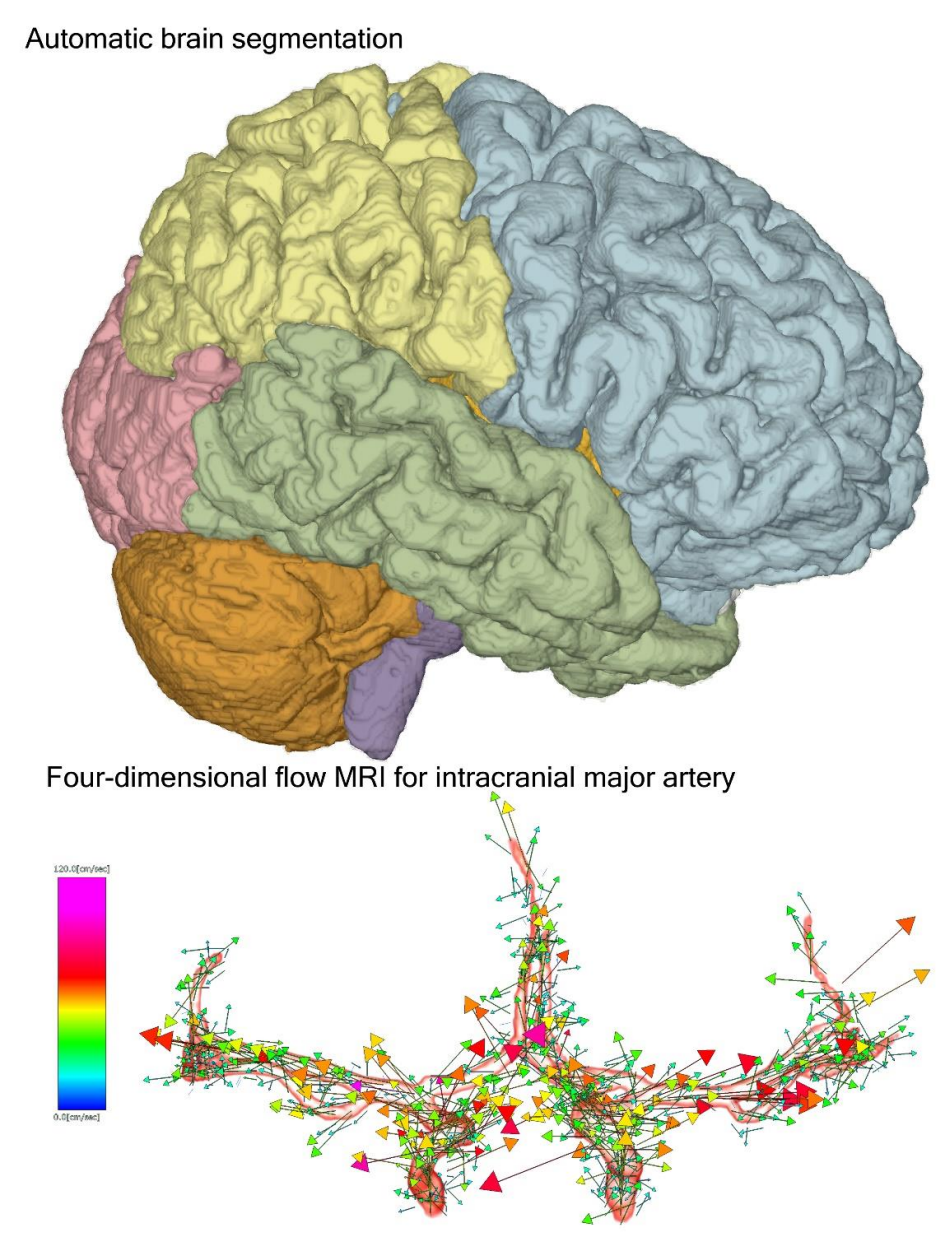
Two applications for 3D workstations used in this study. The upper figure shows the 3D view of automatically segmented brain using Brain Subregion Analysis application. The lower figure shows a 3D vector of flow velocity in the main cerebral arteries around the circle of Willis on the 4D flow application.

## DISCUSSION

We measured CBFs in intracranial arteries around the circle of Willis and regional brain volume ratios in 129 healthy volunteers, ranging from their 20s to >80s, to investigate the effects of aging and sex differences using two methods: a deep learning-based automatic brain segmentation application and a 4D flow application (Fig. 8).

Furthermore, the relationships between regional brain volume ratios and CBFs were comprehensively evaluated in healthy aging brains. The volume ratio of the cortical gray matter linearly decreased with aging and most strongly correlated with the sum of CBFs in the cerebral arteries distal to the circle of Willis, bilateral ACAs (A2), MCAs (M1), and PCAs (P2), rather than those in the cerebral arteries proximal to the circle of Willis, bilateral ACAs (A1), MCAs (M1), and PCAs (P1), or those in the bilateral ICAs and BA. Although no study reported similar findings to ours, it was considered reasonable to assume the CBF distribution and metabolism in healthy participants in nuclear medicine studies, such as ^15^O-labeled water or gas-inhalation positron emission tomography [10, 23], and ^123^I-iodoamphetamine single-photon emission computed tomography [24, 25]. It is common knowledge that regional CBFs at capillary level measured by nuclear medicine studies did not differ between the right and left cortical gray matter and white matter in healthy individuals. Based on these findings of CBFs measured by nuclear medicine studies and the fact that there are few left-right differences in the diameters of these major arteries, it has been thought that there are small left-right differences in CBFs in the cerebral arteries distal to the circle of Willis in healthy individuals. However, the CBFs measured by 4D flow MRI showed a large difference even in cerebral arteries distal to the circle of Willis. Specifically, the left-right difference in CBFs was significantly large in the MCA and ACA (A1). These findings suggest that blood supply varies more between individuals and between left and right than conventionally thought even in healthy young adults, not only through the circle of Willis, but also through the peripheral leptomeningeal anastomosis, and perfusion in the deep white matter and subcortical gray matter from the perforating arteries or superficial cortical arteries. Therefore, the CBF of the intracranial artery should be determined based on the area supplied by the artery rather than its diameter estimating the CBF is difficult with only the artery diameter, and direct measurement of CBF with 4D flow MRI or 2D PC MRI is necessary to further develop our computational simulation model of cerebral circulation [29-31].

**Table 3:** Left-right difference in segmented brain volume ratio and cerebral blood flow.

	Left	Right	Difference	*P* value
Total brain (volume ratio, %)	38.1 ± 1.7	38.0 ± 1.7	0.13 ± 0.49	0.612
Cortical gray matter (%)	16.1 ± 1.0	16.4 ± 1.0	-0.29 ± 0.25	0.026
Subcortical gray matter (%)	0.72 ± 0.04	0.71 ± 0.04	0.01 ± 0.02	0.010
Cerebral white matter (%)	14.4 ± 0.9	14.4 ± 1.0	-0.01 ± 0.20	0.869
Frontal cortex (%)	5.7 ± 0.4	5.9 ± 0.4	-0.24 ± 0.18	<0.001
Parietal cortex (%)	4.0 ± 0.3	4.0 ± 0.3	0.03 ± 0.13	0.439
Temporal cortex (%)	4.1 ± 0.3	4.1 ± 0.3	0.02 ± 0.13	0.625
Occipital cortex (%)	1.9 ± 0.2	2.0 ± 0.2	-0.07 ± 0.15	0.002
Insula cortex (%)	0.43 ± 0.03	0.45 ± 0.03	-0.02 ± 0.02	<0.001
Limbic system (%)	1.3 ± 0.1	1.0 ± 0.1	0.21 ± 0.07	<0.001
Hippocampus (%)	0.23 ± 0.01	0.25 ± 0.02	-0.02 ± 0.01	<0.001
Basal ganglia (%)	0.49 ± 0.04	0.46 ± 0.04	0.03 ± 0.02	<0.001
Cerebellum (%)	4.7 ± 0.4	4.7 ± 0.4	0.07 ± 0.10	0.113
Lateral ventricle (%)	0.84 ± 0.4	0.71 ± 0.4	0.13 ± 0.15	<0.001
ICA flow (mL/min)	342.8 ± 111.1	330.7 ± 115.7	12.1 ± 98.8	0.306
MCA flow (mL/min)	167.1 ± 54.8	185.8 ± 62.3	-18.8 ± 47.1	0.013
ACA-A1 flow (mL/min)	118.3 ± 52.5	100.6 ± 46.7	17.1 ± 63.0	0.010
ACA-A2 flow (mL/min)	96.0 ± 41.2	86.0 ± 35.8	9.95 ± 33.4	0.096
PCA-P1 flow (mL/min)	85.0 ± 35.3	91.5 ± 44.4	-7.87 ± 37.1	0.282
PCA-P2 flow (mL/min)	88.9 ± 39.7	96.1 ± 38.5	-5.31 ± 28.7	0.059
Total outflow #1 (mL/min)	369.5 ± 111.7	382.0 ± 131.4	-12.0 ± 101	0.675
Total outflow #2 (mL/min)	350.0 ± 115.5	365.7 ± 116.0	-11.4 ± 66.1	0.218

Difference, left minus right. P-value, prevalence ratio for the Wilcoxon rank sum test.

Regarding aging and sex differences, total CBFs decreased with age in proportion to decreased cortical gray matter volume, whereas the cerebral white matter had the largest difference in the 40s and had a low correlation with total CBFs. In addition, the greater the cerebral white matter volume ratio, the greater the sex difference in total CBFs. Conversely, the smaller the cortical gray matter volume ratio, the greater the sex difference in total CBFs. We had thought that if the brain volume ratio is the same, the brain volume itself would be larger with a larger intracranial volume, which would proportionally increase CBF, but our results were quite the opposite: the total CBFs were larger in females with smaller intracranial volumes than in males. There was a trend toward greater total intracranial CBF, especially among women in their 40s and younger, who had a larger cortical gray matter volume. In addition, surprisingly, the mean diameters of the ICA and MCA (M1) were reported to be significantly larger in males than in females [32]. In other words, despite smaller arterial diameters, young women had potentially higher CBFs in the cerebral arteries distal to the circle of Willis, compared to men. This finding may be one of the reasons for the approximately twofold higher incidence of cerebral aneurysms and subarachnoid hemorrhage [33, 34], and a threefold higher incidence of migraine headaches [35]. Indeed, multiple sex differences in brain function and structure were reported [36]; however, the relationship between the total and regional CBFs had not been reported.

This study has some limitations. First, the study design was cross-sectional, not a population-based observational cohort study, involving healthy volunteers with wide-ranging ages. Therefore, the applicability of our results to the general population is limited. Essentially, longitudinal assessments should be ideal to prove the decrease in regional brain volume ratios associated with decreased CBFs; however, such studies require a long period and are difficult to conduct using the same high-resolution MRI machine. Above all, 4D Flow MRI is a rapidly evolving imaging technique. Second, the reliability of CBFs measured by 4D flow MRI was not assessed, although flow velocity measurement in intracranial arteries by 4D flow MRI had been well validated and was already in clinical application [11, 19, 26, 28]. Third, a VENC of 120 cm/sec on 4D Flow MRI, while suitable for ICA, may be difficult to accurately measure flow velocities in peripheral arteries with small vessel diameters and slow flow, such as PCoA and ACoA. Finally, even the strongest correlation between the regional brain volume ratio and CBF of the major intracranial arteries was <0.5. This could be due to a wide variation in the blood supply network, with large individual differences.

In conclusion, we examined age and sex differences in the relationship between regional brain volume ratios and CBFs in healthy adults. The total CBFs of the intracranial major arteries distal to the circle of Willis best correlated with the total cortical gray matter volume ratio. Furthermore, the mean total CBFs were greater in healthy women than in healthy men. In addition, significant left-right differences in CBFs of the intracranial major arteries were observed even on the peripheral side of the circle of Willis. Further examination of diseases related to brain volume and cerebral blood flow in relation to aging and sex is needed.

## Supplementary Materials

The Supplementary data can be found online at: www.aginganddisease.org/EN/10.14336/AD.2023.1122

## Data Availability

The MRI data in this study is not available to the community via any open repositories, because the ethics committees have approved the sharing of the MRI data in this research with collaborative institutes and does not allow its being provided to other institutions. The data will be available only on the condition that the ethics committees approve any new participation in the collaborative research.
