# Supplementary material for: Aging and Sex Differences in Brain Volume and Cerebral Blood Flow
Source: Aging Dis. 2024 Oct 1;15(5):2216–29. doi: 10.14336/AD.2023.1122 (PMC11346398; doi:10.14336/AD.2023.1122)
Supplement: Supplementary file 1 — The Supplementary data can be found online at: www.aginganddisease.org/EN/10.14336/AD.2023.1122 [file AD-15-5-2216-s.pdf]

SUPPLEMENTARY DATA

Aging and Sex Differences in Brain Volume and Cerebral Blood Flow

Hiroto Kawano, Shigeki Yamada, Yoshiyuki Watanabe[4], Satoshi Ii, Tomohiro Otani, Hirotaka Ito, Ko Okada, Chifumi Iseki, Motoki Tanikawa, Shigeo Wada, Marie Oshima, Mitsuhito Mase, Kazumichi Yoshida

© 2023. Kawano H et al. Published online at http://www.aginganddisease.org/EN/10.14336/AD.2023.1122

SUPPLEMENTARY DATA

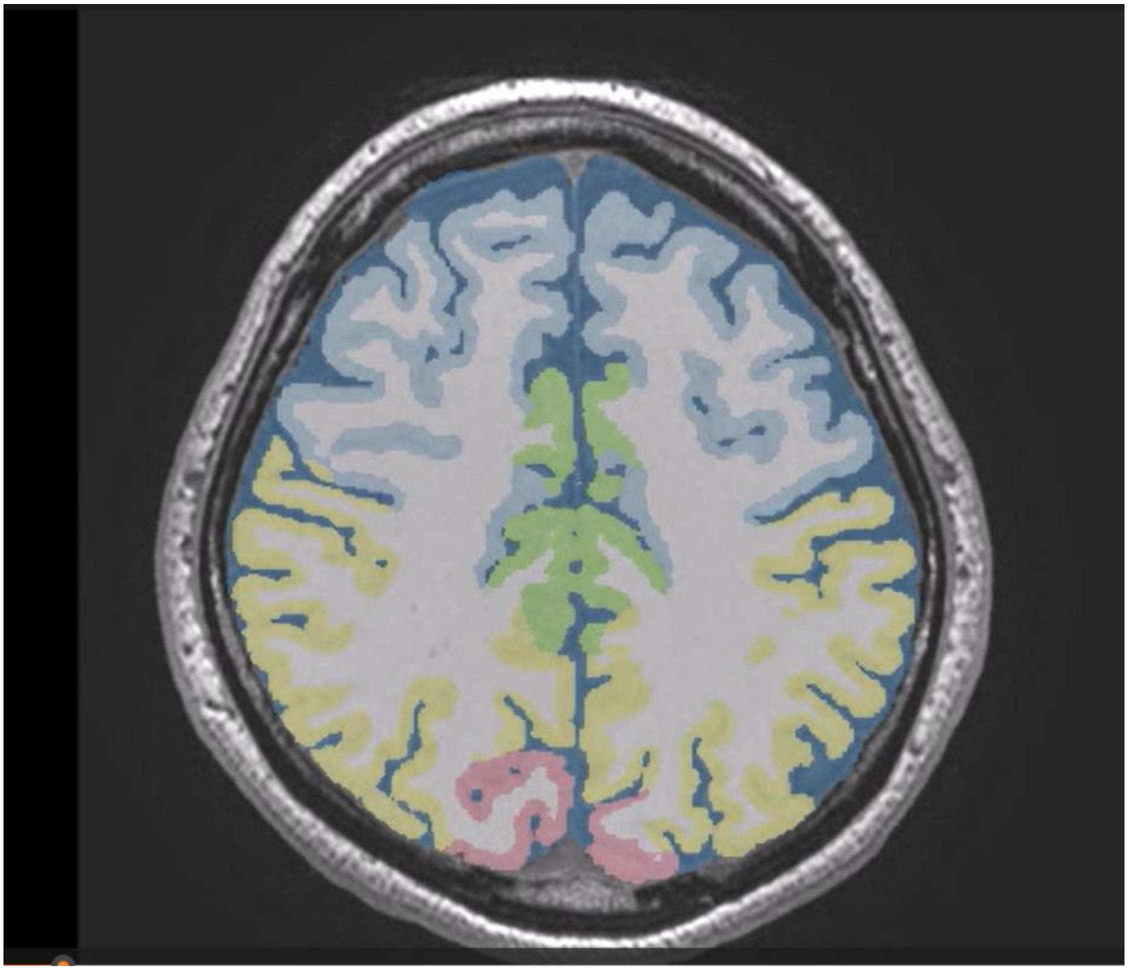

Supplementary Movie 1: The Videoclip shows the sequential images of axial, coronal and sagittal sections of a 48-year-old male healthy brain on the Brain Subregion Analysis application of the 3D volume analyzer SYNAPSE 3D workstation (FUJIFILM Corporation).

© 2023. Kawano H et al. Published online at http://www.aginganddisease.org/EN/10.14336/AD.2023.1122

SUPPLEMENTARY DATA

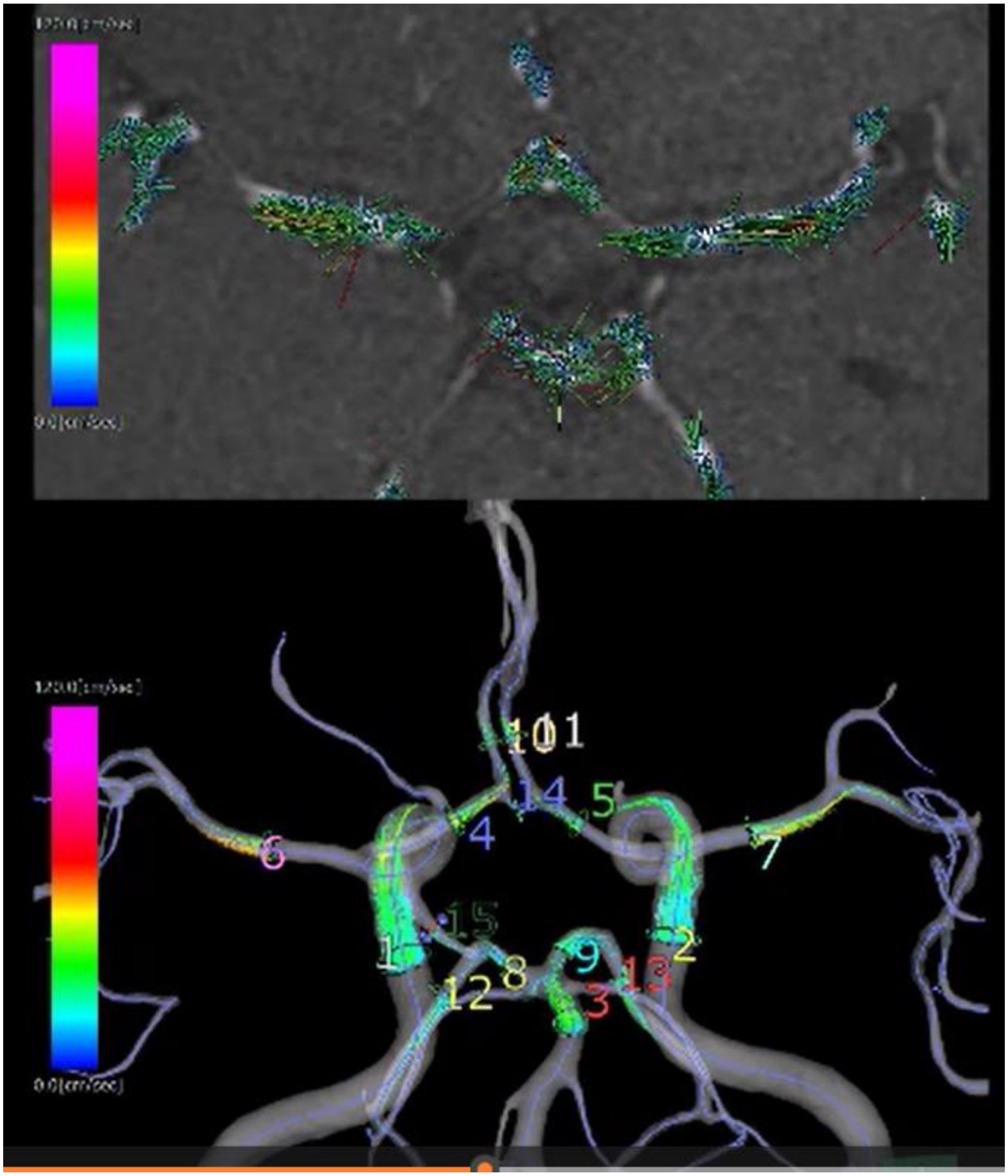

Supplementary Movie 2: The upper videoclip shows the flow vectors and the lower shows the streamlines of the intracranial arteries around the circle of Willis of the same healthy participant as in the Supplement Video 1 (a 48-year-old male) on the 4D flow application of the 3D volume analyzer SYNAPSE 3D workstation (FUJIFILM Corporation).

© 2023. Kawano H et al. Published online at http://www.aginganddisease.org/EN/10.14336/AD.2023.1122

SUPPLEMENTARY DATA

© 2023. Kawano H et al. Published online at http://www.aginganddisease.org/EN/10.14336/AD.2023.1122
